# Comparison of the Characteristics of Small Commercial NDIR CO_2_ Sensor Models and Development of a Portable CO_2_ Measurement Device

**DOI:** 10.3390/s120303641

**Published:** 2012-03-16

**Authors:** Tomomi Yasuda, Seiichiro Yonemura, Akira Tani

**Affiliations:** 1 Plant and Environmental Sciences, Department of Environmental Health Science, University of Shizuoka, 52-1 Yada, Shizuoka 422-8526, Japan; E-Mail: p09402@u-shizuoka-ken.ac.jp; 2 National Institute for Agro-Environmental Sciences, 3-1-3 Kannondai, Tsukuba 305-8604, Japan; E-Mail: yone@affrc.go.jp; 3 Institute for Environmental Sciences, University of Shizuoka, 52-1 Yada, Shizuoka 422-8526, Japan

**Keywords:** multipoint observation, CO_2_ sensor, calibration, portable CO_2_ measurement device

## Abstract

Many sensors have to be used simultaneously for multipoint carbon dioxide (CO_2_) observation. All the sensors should be calibrated in advance, but this is a time-consuming process. To seek a simplified calibration method, we used four commercial CO_2_ sensor models and characterized their output tendencies against ambient temperature and length of use, in addition to offset characteristics. We used four samples of standard gas with different CO_2_ concentrations (0, 407, 1,110, and 1,810 ppm). The outputs of K30 and AN100 models showed linear relationships with temperature and length of use. Calibration coefficients for sensor models were determined using the data from three individual sensors of the same model to minimize the relative RMS error. When the correction was applied to the sensors, the accuracy of measurements improved significantly in the case of the K30 and AN100 units. In particular, in the case of K30 the relative RMS error decreased from 24% to 4%. Hence, we have chosen K30 for developing a portable CO_2_ measurement device (10 × 10 × 15 cm, 900 g). Data of CO_2_ concentration, measurement time and location, temperature, humidity, and atmospheric pressure can be recorded onto a Secure Digital (SD) memory card. The CO_2_ concentration in a high-school lecture room was monitored with this device. The CO_2_ data, when corrected for simultaneously measured temperature, water vapor partial pressure, and atmospheric pressure, showed a good agreement with the data measured by a highly accurate CO_2_ analyzer, LI-6262. This indicates that acceptable accuracy can be realized using the calibration method developed in this study.

## 1. Introduction

Carbon dioxide (CO_2_) is a trace gas in the atmosphere that causes progressive global warming via the greenhouse effect. Since the first observation station for carbon dioxide was established in 1958 at Mauna Loa, Hawaii, the CO_2_ concentration has been measured globally. The CO_2_ concentration is increasing slowly but continuously with a typical seasonal fluctuation.

CO_2_ concentration in urban areas has also been monitored in order to quantify the CO_2_ emission from cities and to investigate the degree of its contribution to the regional carbon budget. Idos *et al.* [1] measured atmospheric CO_2_ concentration in Phoenix (AZ, USA), and found that the concentration at the center of the city (555 ppm) was greater than that in the surrounding rural area (370 ppm). This was attributed to anthropogenic CO_2_ emissions, in particular from vehicular exhaust (79.9%), at the center of the city. They termed the high CO_2_ concentration observed in the central regions of cities as the “urban CO_2_ dome”.

So far, urban CO_2_ has been monitored in many cities, including Chicago, IL, USA [2]; Edinburgh, UK [3]; Marseille, France [4]; Copenhagen, Denmark [5]; Tokyo, Japan [6]; Essen, Germany [7]; Mexico City, Mexico [8]; Basel, Switzerland [9]; and Cairo, Egypt [10]. Most of the urban CO_2_ data were obtained using an observation tower or a vehicle, such as an automobile.

However, the above studies using a few towers or one vehicle provide CO_2_ concentration data only for a small number of fixed points [10] or representative points along the route of the vehicle [7]. A relatively detailed distribution of CO_2_ concentration was obtained by moving a vehicle along various routes [1], but this takes several hours and may not show temporal CO_2_ distribution within a short period of time.

To collect CO_2_ concentration data from many points during a limited measurement period, it is necessary to employ a multi-point observation method using as many fixed stations or observation vehicles as possible. For multi-mobile observation, many CO_2_ sensors should be prepared and therefore, low-cost but accurate CO_2_ sensors are required. If bicycles or electric bikes are to be used for the observations, small and lightweight sensors are preferable.

In recent years, CO_2_ sensors made of semiconductors [11], solid electrolytes [12,13], optic fibers [14], laser diodes [15], and non-dispersive infrared (NDIR) detectors have been developed for monitoring CO_2_ concentration. For atmospheric CO_2_ concentration measurements, NDIR sensors are widely employed since they are stable and very robust against interference by other air components, including pollutants. The NDIR sensor also has excellent durability, and therefore, it seems to be the most reliable sensor for atmospheric CO_2_ measurement [16].

The output of NDIR sensors is affected by temperature, atmospheric pressure, and length of use [17–19]. To enhance the precision and accuracy of NDIR sensors, it is important to correct the sensors’ outputs for these factors. So far, there have been several studies on the calibration of high-cost NDIR analyzers [16–18,20–22]. Only a few studies focused on the performance and calibration methods of small commercial CO_2_ sensors [2,19,23]. There is no report on the recently developed low-cost CO_2_ sensors produced by several manufacturers.

In the present study, to improve the precision and accuracy of recently developed low-cost CO_2_ sensors, a calibration method combining offset-correction and linear correction was examined. On the basis of the results of these performance tests, we have chosen the most suitable CO_2_ sensor for a multi-mobile measurement device. Using this sensor, we developed a portable CO_2_ measurement device that can measure and record temperature, humidity, air pressure, and GPS data as well as CO_2_ concentration. We evaluated the accuracy of this device by measuring the CO_2_ concentration in a school lecture room and by comparing it with the CO_2_ concentration measured by a highly accurate CO_2_ analyzer.

## 2. Experiments

### 2.1. Characterization of CO_2_ Sensors

#### 2.1.1. CO_2_ Sensors Used for Experiments

Five commercial models of diffusion type NDIR CO_2_ sensors were purchased (GMM222C: Vaisala, K30: SenseAir, S100: ELT Co., Korea, AN100: Korea Digital Co. LTD., T6615: GE Sensing & Inspection Technologies). Their measurement ranges are 0–5,000 ppm, except for the GMM222C (0–2,000 ppm). GM222C is about 200 g in weight, 155 mm in probe length, and 18.5 mm in probe diameter, and is used as a reference sensor. The outer dimensions and weight for the other four sensors are less than 82 × 50 mm and 30g, respectively. Detailed information is provided in Table 1. Three individuals for each sensor model were used in the experiment.

#### 2.1.2. Experimental Apparatus and Procedure

**Figure 1.**
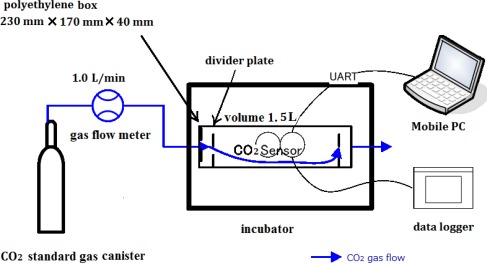
Experimental apparatus.

CO_2_ concentrations of standard reference gases were measured with the individual sensors, and the outputs of each sensor were recorded. Four samples of the prepared standard gas (CO_2_ref_) with CO_2_ concentrations of 0, 407, 1,110, and 1,810 ppm (N_2_-based, Taiyo Nippon Sanso Corporation, Japan) were used. To operate the 13 sensors simultaneously, the individual sensors were arranged in a small box (230 × 170 × 40 mm, 1.5 L) made of polyethylene (Figure 1). This was similar to the Dynamic Enclosure Approach method [23,24]. To control the ambient temperature of the individual sensors, the box was placed in a temperature-controlled incubator (DKM600, Yamato). Universal asynchronous receiver transmitter (UART) cables from the individual sensors were passed through a small hole made on the wall of the incubator and connected to a computer. The output shielded cables of the temperature sensors (LM35DZ, National Semiconductor) and humidity sensors (CHS-UPS, TDK) were also passed outside and connected to a data logger (CR1000, Campbell Scientific). The flow rate of the standard gases into the box was controlled with a flow meter (FS-25CO2, Yamato).

To evaluate the sensors’ output dependency on temperature and length of use, CO_2_ concentrations of standard CO_2_ gases were measured with the individual sensors for different temperatures and durations. Electrical power was supplied to the individual sensors 30 min before the start of the experiment. The standard CO_2_ gas was sent to the sensor box at a flow rate of 1 L/min.

According to the specifications, the response times of all the sensors were less than 2 min. In order to completely replace the air in the small box with standard CO_2_ gas, each standard CO_2_ gas was supplied for 5 min to allow the sensor outputs to stabilize. Thereafter, outputs were recorded every second for 3 min. The sensor outputs against the four standard CO_2_ gases were recorded at different temperatures of 10 °C, 25 °C, and 40 °C on 4 different days (1, 37, 106, and 306) after the start of use.

#### 2.1.3. Cluster Analysis for Classifying CO_2_ Individual Sensors

All the sensor outputs for the standard CO_2_ gases were analyzed by the centroid method of cluster analysis to classify the isolates into different groups. The centroid method uses the notion of the cluster center, defined as the mean vector of the variables for all cases within the cluster. We investigated whether individual sensor outputs had similar patterns between model types and between individual sensors. We drew tree diagrams of the individual sensors by analyzing the outputs obtained at temperatures of 10 °C, 25 °C, and 40 °C on 1 day and 37 days after the start of the use.

#### 2.1.4. Calibration Method of Sensors

Multipoint observation needs many sensors for simultaneous use. All the sensors should be calibrated, but calibrating all the sensors is a time-consuming process. A simple calibration method was examined not only for individual sensors of the same model, but also for each sensor model. In this study, we adopted a combination of zero offset and correction calculation.

First, we measured zero gas and obtained the offset value offset_obs_. The corrected value (CO_2_offset_) was calculated by subtracting offset_obs_ from the sensor output (CO_2_obs_):

(1) $${\text{CO}}_{2\_\text{offset}}={\text{CO}}_{2\_\text{obs}}-{\text{offset}}_{\text{obs}}$$

Temperature dependency of offset_obs_ of the sensors was investigated in advance and we confirmed that offset_obs_ was independent of temperature (10–40 °C) for all the sensor models. Dependency of offset_obs_ on length of use (1–306 days) was also checked and no clear dependency was observed.

Then CO_2_offset_ was corrected for temperature, length of use, atmospheric pressure, and mixing ratio of water vapor (X_w_) using Equation (2):

(2) $${\text{CO}}_{2\_\text{correct}}=K_{\text{std}}\;\;C_{T}\;\;C_{\text{day}}\;\;C_{p}\;\;{\text{CO}}_{2\_\text{offset}}/(1-X_{W})$$

where CO_2_correct_ is the final concentration on a dry air base, and C_T_, C_day_, and C_p_ are correction factors for temperature, length of use, and atmospheric pressure, respectively. K_std_ is the averaged ratio of the three individual sensor outputs measured under standard conditions (25 °C, length of use: 1 day, and 1,013 hPa) to the true CO_2_ concentration of the standard gases.

These factors are linearly expressed by Equations (3–5):

(3) $$C_{T}=1+\alpha_{T}(T-25)$$

(4) $$C_{\text{day}}=1+\alpha_{\text{day}}(\text{day}-1)$$

and:

(5) $$C_{p}=1+\alpha_{P}(P-1013)$$

where T, day, and P are temperature, duration of use, and atmospheric pressure, respectively. α_T_, α_day_, and α_P_ are linear coefficients for C_T_, C_day_, and C_p_, respectively. The terms α_T_ and α_day_ were experimentally determined, but α_P_ was obtained from the sensor instruction manuals.

To determine α_T_ and α_day_, the relative root mean squared error (RRMS error in %) of CO_2_correct_ was calculated using:

(6) $$\text{RRMS error}=\sqrt{\frac{\Sigma {\left(\frac{{\mathrm{CO}}_{2\_\mathrm{correct}}-{\mathrm{CO}}_{2\_\mathrm{ref}}}{{\mathrm{CO}}_{2\_\mathrm{ref}}}\right)}^{2}}{N}}\times 100\;(\%)$$

α_T_ and α_day_ were determined to minimize the RRMS error of CO_2_correct_.

#### 2.1.5. Response Characteristics of Sensor Models

All the CO_2_ sensors used in this experiment were diffusion-type sensors. Because of natural convection in the cells and resistance against air movement caused by a dustproof filter, the output response of the sensors may be delayed after an actual change in CO_2_ concentration. To obtain the response data of the individual sensors, the transient change in sensor outputs was recorded when the CO_2_ concentration was changed from 0 ppm to 407 ppm.

First, standard gas with CO_2_ concentration of 0 ppm was supplied to the sensor box at a flow rate of 1.0 L/min for 10 min to equilibrate the individual sensor outputs. Thereafter, standard gas with CO_2_ concentration of 407 ppm was supplied at the same flow rate for 10 min. We defined t = 0 when CO_2_ concentration was changed from 0 ppm to 407 ppm. The individual CO_2_ sensor outputs were recorded every second, and the individual sensor responses against this drastic change in CO_2_ concentration were investigated.

The sensor output can be expressed in Equation (7) using an offset of time τ and a time constant for the response to equilibrium α. The values are empirically determined for sensors:

(7) $$C_{t}=C_{\text{eq}}\{1-e^{-\alpha (t-\tau )}\}$$

where C_eq_ and C_t_ are the CO_2_ concentrations in equilibrium and at time t (s), respectively.

### 2.2. Experiments Using a Portable CO_2_ Measurement Device

#### 2.2.1. Fabrication of a Portable CO_2_ Measurement Device

**Figure 2.**
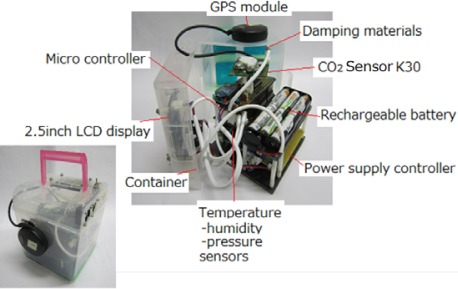
Overview of a portable CO_2_ measurement device.

On the basis of the performance test results described in the Results section, we chose the K30 for developing a portable CO_2_ measurement device (Figure 2). A humidity and temperature sensor unit (SHT-71, Sensirion), a GPS sensor (GPS 18×, Garmin), and an atmospheric pressure sensor (SCP1000-D01, Akitsuki) were incorporated. All data can be automatically recorded on a Secure Digital (SD) memory card (Transcend, 2 GB) under the control of a microcomputer (ATmega2560, Atmel Corporation). The device is powered by 6 lithium-ion rechargeable batteries (eneloop AA cell battery, Sanyo).

#### 2.2.2. Measurement of Indoor CO_2_ Concentration with a Portable CO_2_ Measurement Device

To evaluate the system performance, the portable CO_2_ monitoring device was placed in a room at the Shizuoka Prefectural Science and Technology High School (34.99W, 138.41E) in Shizuoka City, Japan. A widely used and highly accurate CO_2_ analyzer (LI-6262, Licor Co. Ltd.) was also used for comparison. The portable monitoring device and LI-6262 were operated concurrently for a day (2 February 2011) and the data were recorded every 30 s. The CO_2_ analyzer LI-6262 was calibrated with pure N_2_ and CO_2_ standard (407 ppm) gases following the instructions, before the measurement.

## 3. Results

### 3.1. Raw Data Obtained by CO_2_ Sensors

The raw outputs from the CO_2_ sensors for the four different CO_2_ concentrations of the standard gases were, of course, not the same as the standard gas concentrations. The output differences between the sensor individuals of an identical sensor model were less than 54% in K30, but larger (160%) in T6615, suggesting different intra-model variation for different models. In the case of K30 and AN100, their raw outputs increased linearly with CO_2_ concentrations (0.50 < r < 0.99 for K30), but the raw outputs of the other sensors increased nonlinearly (0.103 < r < 0.99). Moreover, the raw sensor outputs for all the models used in this study did not register 0 ppm for pure N_2_ gas, suggesting that offset correction is required.

### 3.2. Cluster Analysis

**Figure 3.**
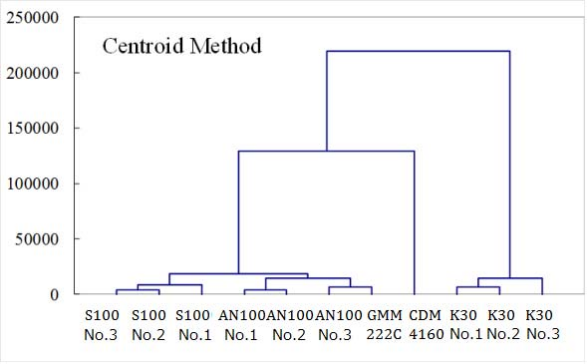
Cluster analysis using the data obtained on 25 February 2010. Air temperatures were 10 °C, 25 °C, and 40 °C, length of use was 1 day. A durable solid electrolyte CO_2_ sensor CDM4160 (Figaro Engineering Inc.) was also included in the analysis.

In our study, cluster analysis was conducted for the output of each sensor (Figure 3). Output patterns differed between sensor models. However, the same tendency was seen in the output patterns of individual sensors in identical models, suggesting that the same calibration method is applicable for the individuals of an identical model, while different calibration coefficients should be determined for different sensor models.

### 3.3. The Calibration Results

A two-point calibration method (combination of offset and span calibration) has been widely used for CO_2_ sensors, including the highly accurate CO_2_ analyzer LI-6262. Since the result of cluster analysis showed that the clustering patterns of individual sensor outputs of the same model were similar, it was proposed that the same method can be applied to calibrate individual sensors of the same model.

Since the CO_2_ standard gas was a mixture of dry air and CO_2_, X_W_ can be assumed to be zero and Equation (2) can be modified as follows:

(8) $$C_{T}C_{\text{day}}={\text{CO}}_{2\_\text{std}}/K_{\text{std}}C_{p}{\text{CO}}_{2\_\text{offset}}$$

where CO_2_std_ is CO_2_ standard gas concentration.

**Figure 4.**
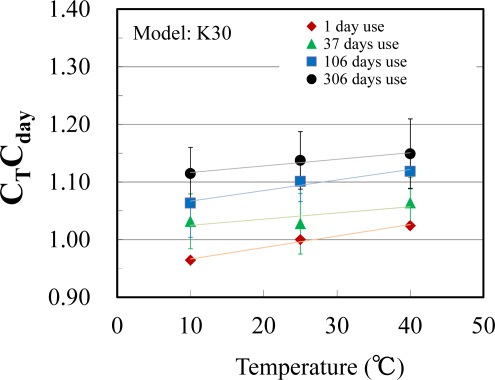
Relationship between temperature and C_T_C_day_ in the case of K30 (n = 3).

C_T_C_day_ was calculated using Equation (8) for each measurement at a different temperature and for different length of use. C_T_C_day_ was plotted against temperature and length of use, and the relationships between them were investigated. Figure 4 shows the relationship between C_T_C_day_ for K30 and T. C_T_C_day_ increased linearly with increasing T, and the values of the slope were not greatly different among data obtained for different lengths of use. Linear relationships were not observed in the case of AN100 and S100.

**Figure 5.**
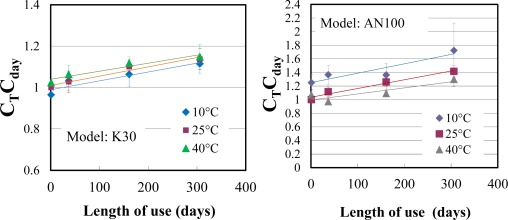
Relationship between C_T_C_day_ and length of use in the case of K30 and AN100 (n = 3).

**Figure 6.**
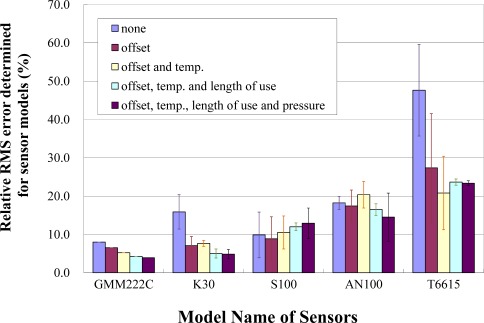
Average RRMS errors using coefficients determined for sensor models.

The relationship between C_T_C_day_ and length of use (Figure 5) shows the linearity in the case of K30 and AN100. α_T_ and α_day_ were determined to minimize the RRMS error between CO_2_std_ and CO_2_correct_ obtained from Equation (2). RRMS errors calculated using α_T_ and α_day_ determined for individual sensors and for sensor models are listed in Table 2. RRMS errors determined for individual sensors were lower than those determined for sensor models, and the difference ranged from 4.8% to 23.6%. RRMS errors decreased in both cases with an increase in the number of correction factors considered in the K30 and AN100 sensor models, suggesting that these corrections successfully improve the accuracy of the sensors. In particular, K30 had the highest accuracy, comparable to the reference sensor GMM222C, even when the same coefficients were applied to three sensors of the K30 model (Figure 6). This result suggests that the coefficients determined for sensor models using all the data from three individual sensors can be applied for sensor correction for the K30 model.

RRMS errors determined for S100 and T6615 were increased with increasing calibration parameters (Table 2). Since the raw outputs of these sensors did not increase linearly with temperature and length of use, the calibration method might not be adequate for these sensor models.

### 3.4. Response Time of the Sensors

**Figure 7.**
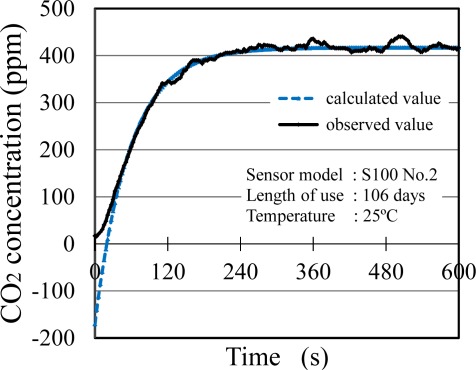
Time course of the output of S100 sensor model. Solid line shows actual sensor output value. Dotted line shows CO_2_ concentration estimated using α and τ.

It is necessary to consider the response delay of the sensor output values against actual CO_2_ concentration change. Figure 7 shows the response of the output of the S100 sensor model. A time constant for the response to equilibrium α and an offset of time τ in response of each sensor model were calculated from Equation (7) (Table 3). Each sensor had a good agreement between the calculated curve fit and actual data when τ in response was employed. The 90% response time of all the sensors, excluding T6615, was <3 min, and these values were all larger than the catalog values. The responses of small CO_2_ sensors used in this study were slower than that of the reference sensor GMM222C. No clear relationship between the length of use and the sensor response was observed. No temperature dependency of α and τ was also confirmed (data are not shown).

### 3.5. Development of a Portable CO_2_ Measurement Device and Validation Test

The developed measurement device was compact (100 × 100 × 150 mm) and lightweight (900 g). Measured data can be displayed on a graphic 2.5 inch liquid crystal display in real-time. The device is powered by six lithium-ion AA batteries and can be operated for 5 h continuously. An observation interval and the coefficients required for the CO_2_ calibration can be input into an on-board microcomputer. The cost of production was about 60,000 yen or USD 770 per device.

The CO_2_ concentration in a lecture room of the Shizuoka Prefectural Science and Technology high school was measured every 30 s throughout a day (7 February 2011) using a high-precision CO_2_ analyzer (LI-6262) and the developed portable CO_2_ measurement device (Figure 2). Before the measurement, the portable CO_2_ monitoring device was corrected for offset value using the pure N_2_ gas. The sensor output of the portable CO_2_ measurement device was corrected for temperature, length of use, atmospheric pressure, and water vapor partial pressure.

**Figure 8.**
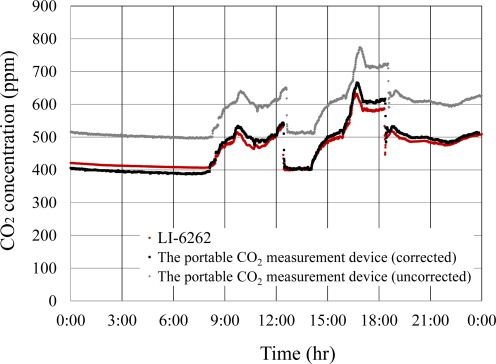
Comparison of CO_2_ concentration measured with the portable CO_2_ measurement device and LI-6262 on 7 February 2010.

Owing to the response delay of the sensor in the developed device, the measured CO_2_ data had to be time-shifted to the CO_2_ data obtained with the LI-6262. Subsequently, the RRMS difference of the portable CO_2_ measurement device against the LI-6262 was calculated. The RRMS difference was 3.5%, indicating a good agreement of the outputs between the high-precision CO_2_ analyzer and the developed device (Figure 8).

### 3.6. Discussion

A simple 2-point calibration method is widely used for many sensors, including CO_2_ analyzers. Other calibration methods such as a second-order polynomial equation [20] and a cubic spline function [25] have been applied in the case of CO_2_ sensors. In this study, the K30 and AN100 showed a good linear relationship between the sensor outputs and standard CO_2_ gas concentrations. These clear linear relationships were observed under most measurement conditions for different temperatures and lengths of use. The other two sensor models showed no clear relationship between the standard CO_2_ gas concentrations and their outputs.

When many CO_2_ sensors are used for a spatial distribution measurement of CO_2_ concentration within a limited time, the calibration of each sensor is a time-consuming process. A simplified calibration method is required. Pandey *et al.* compared CO_2_ data obtained with all sensor units manufactured by the same company, and found an excellent compatibility between them throughout the entire side-by-side analysis [3]. In our measurements, we found similar tendencies for individual sensor outputs within identical sensor models by cluster analysis. We also obtained acceptable RRMS errors, even when the coefficients for temperature and length of use were determined using all the data obtained from the three sensors of the same model. We have chosen the K30 sensor model to minimize RRMS errors and propose that, in practical measurements, only the offset calibration shown in Equation (1) should be conducted and then CO_2_correct_ is calculated using α_T_ (=0.00141) and α_day_ (=0.000478) determined here. Our result suggests that, once the coefficients for the sensor model are determined, the coefficients can be applied for other individual sensors of the same model. This greatly reduces the effort required for calibration compared with conventional calibration.

The 90% responses of the CO_2_ sensors used in this study were slower than that of the reference sensor GMM222C (Table 3). This is probably caused by gas diffusion from one side only in the box-type IR cells of all the sensors compared with diffusion from both sides in the cylinder-type IR cell of GMM222C. The thickness and material of the dustproof filter of the sensors might also provide a resistance for gas diffusion.

Time drift [18] and temperature dependency [21] of the IR detector in CO_2_ sensors were reported to be important factors to be considered. Sega *et al.* [20] confirmed that there was little difference in the secondary polynomial curve for CO_2_ outputs of an analyzer observed for lengths of use of 1 month and 3 years. They proposed that the calibration of the analyzer should be conducted once a year. Apart from the above study, there are no reports investigating effect of the length of use on the output of CO_2_ analyzers. In this study, we found that K30 has a linear relationship with length of use and showed that the slope can be used to correct the sensor output for length of use. However, it should be noted that the coefficients determined in this experiment may not be valid if the sensor is used for over 1 year.

Our results indicate that temperature, pressure, and length of use independently affect the output of the sensors, and therefore, the calibration equation can be expressed as a product of correction terms for the three parameters. The RRMS error was lowered by using the three independent correction terms. When using several sensors simultaneously, the difference in the sensor outputs might result in significant measurement errors. We found that the sensor output tendency against environmental factors was similar between sensor individuals of the same sensor model, but different between sensor models. On the basis of this result, we could propose a method that corrects the outputs of several sensors of same model by using one coefficient each for temperature, air pressure, and length of use. This can eliminate the time-consuming process of calibration and reduce the sensor output error caused by using several sensors simultaneously for CO_2_ distribution measurement.

In recent years, CO_2_ measurement techniques based on on-road mobile laboratories have been applied in urban areas to determine the typical inhomogeneous spatial distribution of CO_2_ concentration and emission sources within urban areas [1,7,26–30]. Most of the mobile laboratories were mounted on a car, and therefore, the measurement area is limited to roads. However, one can easily carry the portable CO_2_ measurement device developed in this study in a hand and measure CO_2_ concentration not only by using a vehicle but also by walking around with this device since it is lightweight and battery-operated. CO_2_ concentration, temperature, humidity, atmospheric pressure, and measurement time and location can be measured and recorded onto an SD memory card. The portable CO_2_ measurement device can be used to measure the CO_2_ concentration in urban forests, public greens, and industrial areas for scientific research and environmental education purposes.

## 4. Conclusions

When four samples of standard gas with different CO_2_ concentrations (0, 407, 1110, and 1810 ppm) were measured with four models of small commercial CO_2_ sensors, the outputs of the K30 and AN100 systems showed linear relationships with temperature and length of use. With an increase in the number of environmental factors considered for calibration, the accuracy of K30 and AN100 was improved. In particular, the accuracy of the K30 improved significantly, even when the same correction coefficients were used for three individual sensors of this model. Using the K30, we have developed a portable CO_2_ measurement device. Good agreement was obtained for the outputs between a high-precision CO_2_ analyzer and the developed device. This portable device allows measurements to be made while walking and cycling. The measurement device can be used for measuring the heterogeneity of CO_2_ distribution in urban areas and for environmental education.

## Figures and Tables

**Table 1.** Catalog specifications of five commercial diffusion type NDIR CO_2_ sensors.

**Sensor**	**GMM222C**	**K30**	**S100**	**AN100**	**T6615**
manufacturer	Vaisala a cylinder with 18 mm diameter and 140 mm length *	SenseAir	ELT	KCD	GE sensing
L×W×D (mm)		51 × 57 × 14	33 × 33 × 13	82 × 45 × 18	57 × 35 × 15
Weight (g)	220	17	10	29	17
Measurement range (ppm)	0–2,000	0–5,000	0–5,000	0–5,000	0–5,000
Accuracy	30 ppm + 2% of reading	30 ppm + 5% of reading	30 ppm +5% of reading	200 ppm + 3% of reading	75 ppm or 10% of reading
Response Time (s)	30 (63%)	20 (63%)	60 (90%)	30 (63%)	<120 (90%)
Operating voltage (V)	11–20 VDC	4.5–14 VDC	5.0–5.5 VDC	8–14 VDC	5 VDC

* Size of a probe housing.

**Table 2.** Average RRMS errors using coefficients for temperature, length of use, and atmospheric pressure.

**Sensor Model**	K30		S100		AN100		T6615	

	Individual Coefficient*	Model Coefficient **	Individual Coefficient *	Model Coefficient **	Individual Coefficient *	Model Coefficient **	Individual Coefficient *	Model Coefficient **
**temperature**	7.3	7.7	9.6	10.5	13.0	20.4	20.5	20.8
**temp + day**	4.5	5.0	11.1	12.0	15.3	16.5	18.3	23.6
**temp + day + pressure**	4.4	4.8	11.5	12.9	14.5	14.6	17.3	23.3

* The average RRMS error determined using coefficients determined for individual sensors.

** The average RRMS error determined using coefficients determined for sensor models. Same coefficients were used for the calibration of three sensors.

**Table 3.** Offset of time τ, time constant for the response to equilibrium α and 90% response time for sensors.

**Sensor Model**	**GMM222C**		**K30**		**S100**		**AN100**		**T6615**	
**Length of Use (days)**	37	306	37	306	37	306	37	306	1	200
**α (s^−1^)**	0.055	0.014	0.045 ± 0.026	0.024 ± 0.004	0.014 ± 0.003	0.038	0.015 ± 0.004	0.019 ± 0.006	0.0215 ± 0.004	0.014 ± 0.003
**τ (s)**	5.7	2.6	6.1 ± 1.8	4.8 ± 2.6	12.7 ± 1.2	16.7	13.5 ± 3.7	14.5 ± 7.4	14.2 ± 10.0	19.4 ± 18.0
**90% Response Time (s)**	47.5	87.8	87.8 ± 52.1	102.9 ± 18.9	175.3 ± 38.2	77.8	151.0 ± 28.1	141.6 ± 39.2	124.2 ± 26.2	190.4 ± 38.8

Measured at 25 °C, n = 3.
